# Unusual Origin of the Posterior Circumflex Humeral Artery: A Case Report

**DOI:** 10.7759/cureus.36316

**Published:** 2023-03-17

**Authors:** Norris C Talbot, James V D'Antoni, Lenise G Soileau, Nicholas R Storey, Adegbenro Fakoya

**Affiliations:** 1 Cellular Biology and Anatomy, Louisiana State University Health Sciences Center, Shreveport, USA

**Keywords:** surgery, quadrangular space, anomaly, variation, subscapular artery, posterior circumflex humeral artery

## Abstract

Prior knowledge of possible variations in human anatomy is essential for basic medical and clinical training. Many surgeons can avoid uncharacteristic situations by having sources and availability of resources that document potential irregularities in human anatomy. In this case, a human cadaver is identified as having an altered origin of the posterior circumflex humeral artery (PCHA). While it usually stems from the axillary artery, this cadaver had a left-sided PCHA originating from the subscapular artery (SSA) and continuing into the quadrangular space. This irregularity of the PCHA from the SSA is not commonly discussed in the literature. Physicians and anatomists need to be fully aware of this possibility and be prepared for any unexpected differences in anatomy during procedures.

## Introduction

The posterior circumflex humeral artery (PCHA) is an important blood vessel due to its massive responsibility in supplying the head of the humerus, glenohumeral joint, and shoulder muscles [1]. Usually, the PCHA will originate directly from the axillary artery, which is divided into three segments. The first artery, commonly off the axillary artery, is the superior thoracic artery, originating from the first segment [2,3]. The second segment has both the thoracoacromial artery and the lateral thoracic artery. The third segment of the axillary artery commonly includes the subscapular artery (SSA), the anterior circumflex humeral artery (ACHA), and PCHA [3]. The SSA, the largest branch of the distal part of the axillary artery, usually divides into the thoracodorsal and circumflex scapular arteries. The ACHA and PCHA will typically branch off the axillary artery into the quadrangular space along with the axillary nerve [3].

The PCHA usually arises from the third part of the axillary artery distal to the pectoralis minor muscle, proximal to the border of the teres major muscle, and posteriorly to the ACHA, which will usually follow it [1}. Piercing through the quadrangular space, the PCHA follows the path of the axillary nerve [4].

Anatomical anomalies of the origin of the PCHA are considerably rare. Some documented studies exist, such as the PCHA branching from the deep brachial artery, emergence from a common trunk in the second section of the axillary artery, and branching into the triangular space from the SSA [5-7].

The prevalence of PCHA from the SSA is infrequently documented despite multiple implications in upper limb neurovascular pathology and expectations in surgical procedures. A study reported the prevalence of this anomaly as one in 10 cadavers analyzed [8]; however, this anomaly may occur in up to 20% amidst other described variants [1]. In this case report, we will show the unusual occurrence of a PCHA from the SSA into the quadrangular space and highlight the clinical implications of this anomaly.

## Case presentation

**Figure 1:**
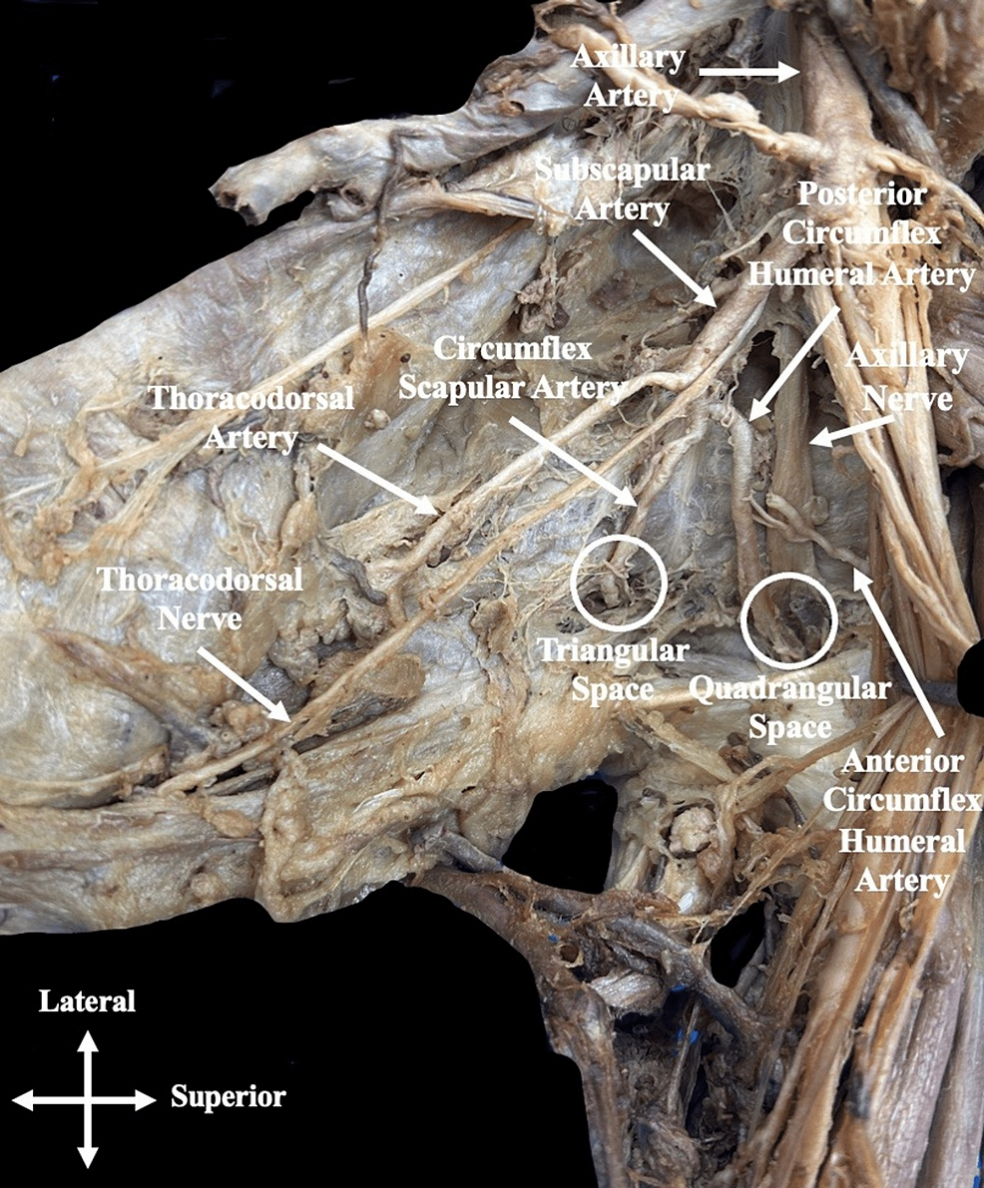
Anterior view of the left axilla region An anterior view of the left axilla region after exposing the axillary artery and associated branches. Here the posterior circumflex humeral artery arises as a third branch from the subscapular artery. Moving laterally, it joins the axillary nerve to enter the quadrangular space.

**Figure 2:**
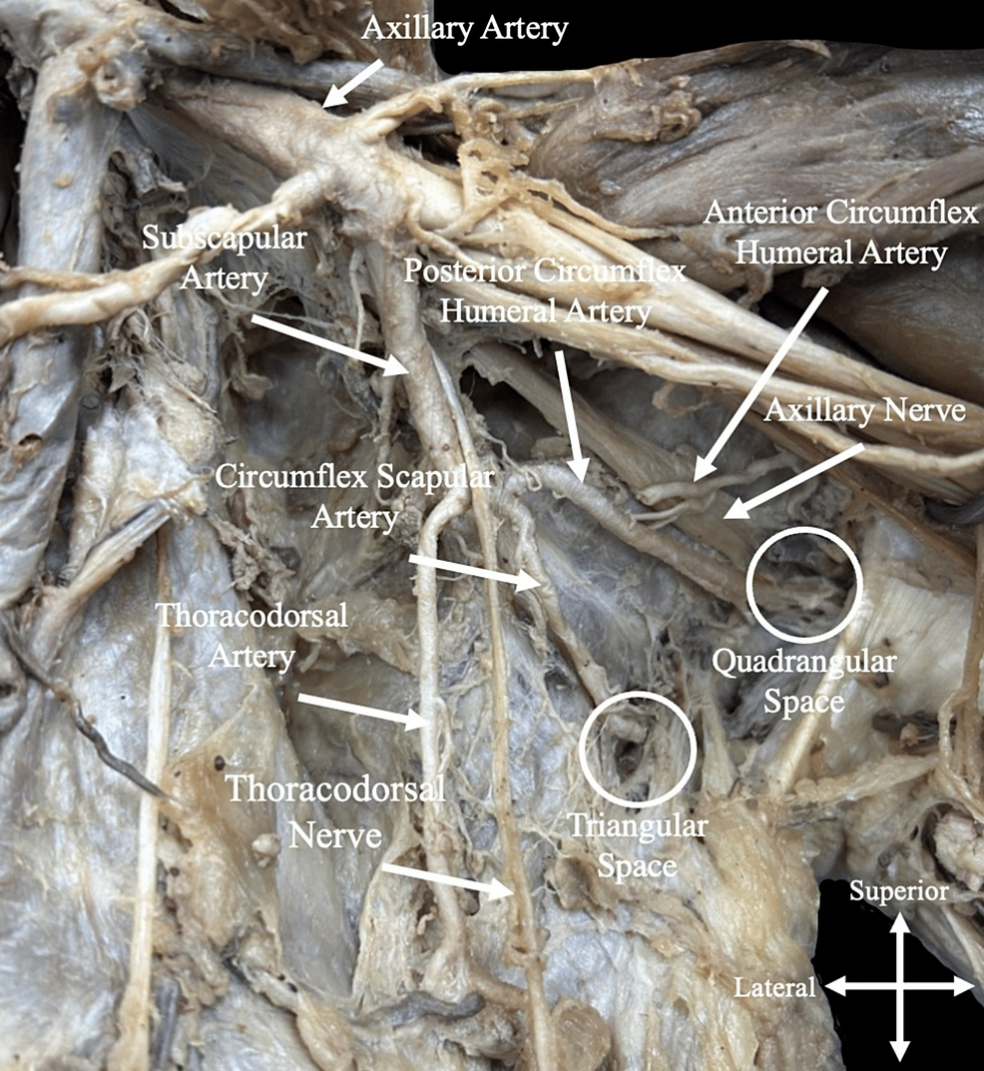
A targeted anterior view of the subscapular artery. A targeted view of the subscapular artery and its three branches: the thoracodorsal artery (supplying the latissimus dorsi), the circumflex scapular artery (entering the triangular space), and the posterior circumflex humeral artery (entering the quadrangular space).

Medical students at Louisiana State University Health Sciences Center, Shreveport School of Medicine, Louisiana, United States, discovered an anomalous branching of the left PCHA during a routine cadaveric dissection of a 78-year-old Caucasian male. In this case, the SSA was identified in the left axilla as branching inferiorly from the axillary artery and distally giving rise to both the circumflex scapular artery and thoracodorsal artery, as is typical. However, the PCHA was also identified as an equally distal branch off the SSA at a position lateral to the circumflex scapular and thoracodorsal arteries, as shown in both Figure 1 and Figure 2. The PCHA was then observed to proceed laterally and enter the quadrangular space inferior to the axillary nerve.

Notably, the ACHA was believed to have been cut in the dissection and could not be traced back to the axillary or subscapular artery. Otherwise, the structure and course of the axillary artery and its branches were unremarkable in the right axilla.

## Discussion

**Figure 3:**
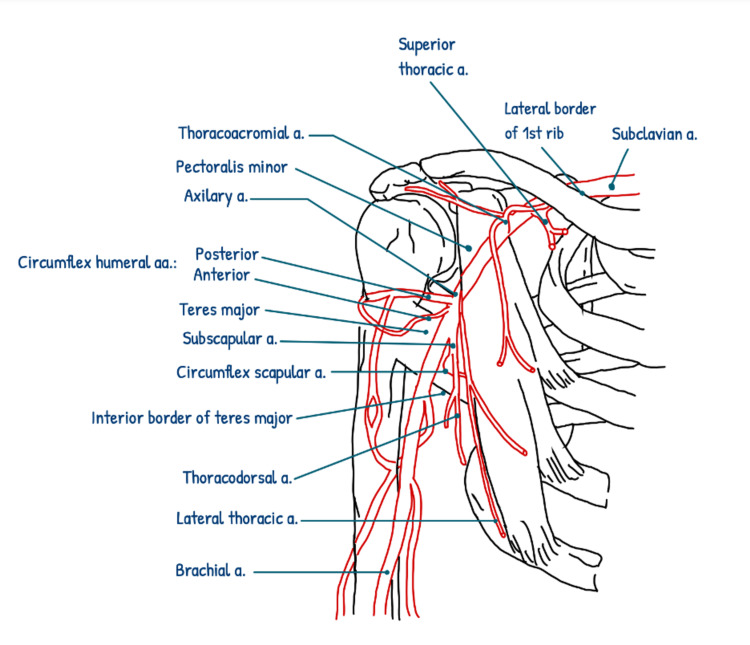
Schematic diagram of the axillary artery illustrating the normal origin of the posterior circumflex humeral artery from the distal part of the axillary artery. a: artery

The axillary artery exists as the continuation of the subclavian artery once it crosses the border of the first rib [9]. The axillary artery becomes the brachial artery after crossing the inferior margin of the teres major muscle [10]. Most often, the axillary artery will be defined by three segments relative to the pectoralis minor muscle. The first segment is proximal to the pectoralis minor and has one branch, the second is deep to the pectoralis minor and has two branches, and the third is distal to the pectoralis minor and has three branches [9]. This is shown in Figure 3. 

In this dissection, the PCHA was clearly visualized entering the quadrangular space but originating from the SSA. Noted was also the absence of atypical arteries or branchings from the axillary artery. The occurrence of the PCHA from the SSA has been infrequently documented despite the implications that might exist for different surgical procedures.

Understanding and documenting cases of anatomical irregularities of PCHA is especially important for anatomical dissections, generating sound clinical advice and surgical protocols. The PCHA has a few significant clinical implications that could be further complicated when considering the potential for its irregular origin from the subscapular artery. Quadrangular space syndrome, often associated with repetitive trauma or overhead activities (volleyball and baseball), may cause impingement of the PCHA and axillary nerve leading to atrophy and weakness of the deltoid and teres minor muscles. [11]. Additionally, hemodynamic implications should be considered in cases of PCHA rupture among individuals with this anomaly. We hypothesize that rupture of the PCHA under these circumstances could have devastating consequences as circulation from the circumflex scapular and thoracodorsal arteries supplying the latissimus dorsi and various scapular muscles could be diminished, leading to ischemia.

The PCHA arising from the SSA also has surgical implications. Surgeries dealing with the anatomical reduction of a fracture in the proximal humerus may use predetermined landmarks to access the PCHA quickly. A recent study identified these landmarks and reported the mean distance from its origin at the third part of the axillary artery to the infra glenoid tubercle, coronoid process, acromion process, and midclavicular line were 27.7 mm, 50.2 mm, 68.4 mm, and 75.8 mm, respectfully [12]. It is essential to understand that these landmarks may differ among individuals whose PCHA arises from the SSA. Hence, an anthropometric study might be needed to determine the landmarks for a PCHA originating from the SSA. Furthermore, understanding this irregularity for other uncommon surgeries, such as axillary tissue flap formation and axillary scar release, may benefit surgeons [13]. While surgeons must have a firm grasp of basic anatomy often presented in textbooks, anatomical irregularities are extremely common and should also be studied. Knowledge of common neurovascular irregularities in the axillary region and proper preoperative radiologic imaging is vital for successful surgical outcomes [14].

## Conclusions

The PCHA provides a critical blood supply to the proximal humerus and surrounding shoulder muscles, and the anomalous anatomy of the region must be understood in order to properly treat conditions of the axillary region, especially in a surgical setting. This atypical finding involving the PCHA has been infrequently described in anatomical literature despite the potential importance of proper identification by radiologists, surgeons, and other providers. Further investigation into the frequency of this and similar variations is recommended.
